# Prevalence and pathogenesis of some filarial nematodes infecting donkeys in Egypt

**DOI:** 10.14202/vetworld.2016.888-892

**Published:** 2016-08-22

**Authors:** A. M. Radwan, N. E. Ahmed, L. M. Elakabawy, M. Y. Ramadan, R. S. Elmadawy

**Affiliations:** Department of Parasitology, Faculty of Veterinary Medicine, Banha University, Moshtohor, Toukh 13736, Egypt

**Keywords:** age, donkey, Filaria, gender, prevalence

## Abstract

**Aim:**

The primary objective of the present study is to determine the commonness of filarial parasites in donkeys in Egypt, identification of the filarial species tainting them and the delivered pathogenic impact connected with the infestation.

**Materials and Methods:**

A total of 188 donkeys were examined for filarial infection. The blood samples and scraping of the cutaneous bleeding lesions were collected, stained, and inspected for microfilariae all through the period from March 2011 to October 2013. The adult worms were perceived in tissue samples acquired from skin scraping, testes, eyes, tendons, peritoneal and pleural cavities, and the ligamentum nuchae.

**Results:**

On the basis of morphological identification, 163 of 188 donkeys (86.70%) were infected with *Onchocerca cervicalis* (82.98%), *Setaria equina* (31.11%), *Parafilaria multipapillosa* (5.32%), and *Onchocerca reticulata* (4.26%). There was no significant effect of the sex on the incidence of all the encounteredfilarial worms except for *S. equina*, where the infection rate prevailed in males versus females (40.82% vs. 35.90%). In addition, age group of 5-15 years old exhibited a fundamentally higher predominance (p< 0.05) of the recognized filarial worms versus those of < 5 years old and >15 years old.

**Conclusion:**

The preliminary results add to our comprehension of filarial species infecting donkeys in Egypt, their impact on animal execution and production. Accentuation must be taken for avoidance, control of filarial disease, and improvement of the management system of donkeys.

## Introduction

In Egypt, Donkeys, *Equus asinus*, are widely spread and economically important animals used for transport, whether riding, pack transport, or pulling carts. They provide more prominent mobility with which to face erratic rainfalls and are of worth in conveying firewood, loads, including water, household structures, goods, and children [1]. Filariasis is one of the most critical parasitic sicknesses affecting equines in Egypt. It is caused by different filarial species, namely, *Onchocerca* sp., *Setaria equina*, and *Parafilaria multipapillosa*. Substantial filaria infection in donkey is mainly associated with a destructive effect, including debilitation and limitation of the animal movement due to either affection of the ligamentum nuchae by *Onchocerca cervicalis* or affection of the flexor tendons and the suspensory ligaments of the forelegs by *Onchocerca reticulata* [2]. A severe inflammatory reaction is produced around the dead worm, surrounded by fibrous tissue and the worm become calcified which may encourage the secondary bacterial invasion and abscess formation. Moreover, the filarial worms may migrate to unusual habitats such as ocular glob causing ocular dermatitis [3] and central nervous system causing serious pathogenic impacts [4]. Cases of eye infection with *Setaria equina* have been reported in human by Taylor *et al*. [5]. Whereas, human infection with *Onchocerca* sp. is associated with firm subcutaneously located nodules in the tendons of the muscles, knees, wrists, or feet [6]. In Egypt, there are few reports dealt with filariasis in donkeys, their prevalence or pathogenicity. Hence, the plan of the present study is to determine the commonness of filarial parasites in domestic donkeys (*E. asinus*) in Egypt, identify filarial species tainting them, assess the impact of sex and/or age on their prevalences, and to recognize the delivered pathogenic impact connected with the infestation.

## Materials and Methods

### Ethical approval

The collected samples in the present study were approved by the local committee of the Faculty of Veterinary Medicine, Benha University and according to the guidelines of the National Institute of Health in Egypt.

### Animals

The study was conducted on 188 donkeys that were submitted for slaughtering, and their meat was utilized as meals for wild animals in Giza zoo. Each donkey was physically examined before slaughtering for clinical signs or lesions created by filariasis.

### Samples

For standard microscopic examination of microfilariae, 10 ml of blood was collected from the jugular vein of each donkey in the early morning at 7- 8 am into dry elastic stoppered glass tubes containing 0.1 g ethylenediaminetetraacetic to be utilized for wet blood and stained thick blood smears preparations. Microfilariae were likewise perceived in scraping of presenting cutaneous bleeding lesions and by skin snip biopsy (measuring 2 cm × 2 cm) of the midline of the abdomen and umbilical region using cattle ear punch. The tissues were minced, suspended in 10 ml distilled water, and incubated overnight at room temperature. The released microfilariae were concentrated by centrifugation at 500 rpm for 10 minutes. The sediment was examined under microscope (×10) for the movement of the microfilariae, then they were fixed with 2% formalin and stained by Giemsa stain or methylene blue 1:1000 for identification.

The adult worms were acquired from various tissues of the slaughtered donkeys. Ligamentum nuchae were removed, preserved in ice, dissected by peeling apart its two layers and the visible part of the *O. cervicalis* was gently grabbed. Flexor and extensor tendons and suspensory ligament were also inspected carefully for *O. reticulata*. Furthermore, peritoneal cavities, pleural cavities, scrotums, and eye were checked for the presence of *P*. *multipapillosa* or *S. equina*. Preparation of permanent mount of adult worm was done, and the morphological features of the filarial worms were distinguished as described by Soulsby [2].

### Gross pathology

Fresh tissues were transported to the laboratory, and they were dissected using a stereo microscope for detection of the pathological changes associated with filarial nematodes.

### Statistical analysis

Statistics was executed using two ways ANOVA under significance level of 0.05 for the entire results using SPSS (version 19).

## Results

**Figure-1:**
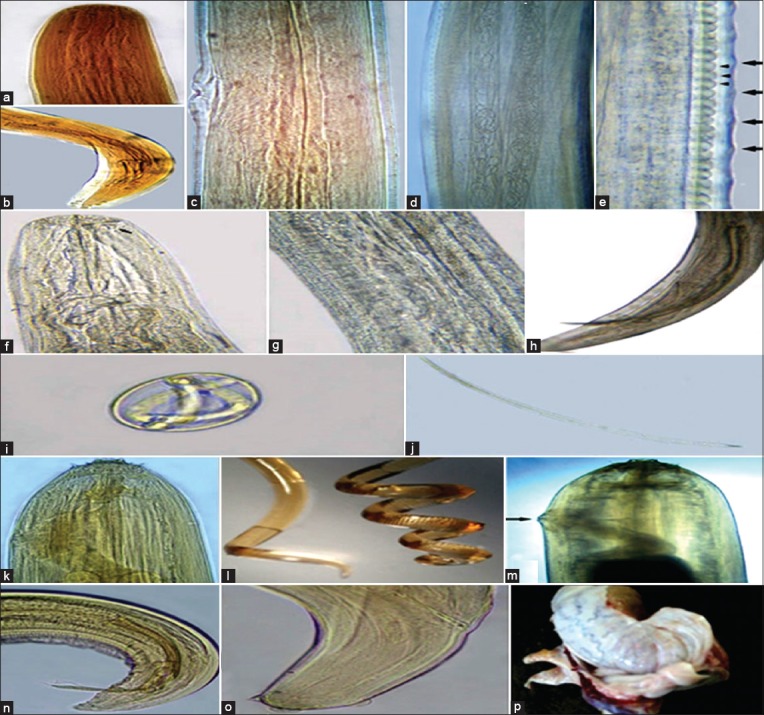
*Onchocerca cervicalis*: (a) Anterior end, (b) male posterior end, (c) vulva, (d) microfilariae in the uterus of female (e) middle region of female showing low widely spaced indistinct external cuticular annulations (large arrows) and internal striations forming elongated cells (small arrowheads); *Parafilaria multipapillosa*: (f) Anterior end (arrow refers to vulva anteriorly near the mouth opening), (g) Uteri in the middle region, (h) male posterior end, (i) embryonated egg with thin flexible shell, (j) microfilaria with rounded posterior extremity; *Setaria equina*: (k) Anterior end, (l) coiled male posterior end (right) and female (left), (m) female vulva, (n) male posterior end, (o) female posterior end, (p) adult worm encapsulated between the testicular tunica.

In the present survey, the evidence of filarial infection was experienced in 86.70% out of 188 inspected donkeys. *O. cervicalis* was the most abundant species (82.98%) as compared with *S. equina*, *P. multipapillosa*, and *O. reticulata* (36.17%, 5.32%, and 4.26%, respectively) (Table-1 and Figure-1).

**Table-1:** Effect of gender on the prevalence of filarial nematodes.

Gender	Number infected (%)	Infection (%)			*P. multipapillosa*

		*O. cervicalis*	*O. reticulata*	*S. equina*	
Males (n=98)	85 (86.73)^aA^	80 (81.63)^aA^	5 (5.10)^aC^	40 (40.82)^aB^	4 (4.08)^aC^
Female (n=90)	78 (86.67)^aA^	76 (84.44)^aA^	3 (3.33)^aC^	28 (31.11)^bB^	6 (6.67)^aC^
Total (n=188)	163 (86.70)	156 (82.98)	8 (4.26)	68 (36.17)	10 (5.32)

Different superscripts letters

(a,b,c) in the same column indicate significant differences at p<0.05. Different superscripts letters

(A,B,C) in the same row indicate significant differences at p<0.05. *O. cervicalis=Onchocerca cervicalis, S. equina=Setaria equina, O. reticulata=Onchocerca reticulate, P. multipapillosa=Parafilaria multipapillosa*

No significant effect of the sex on the incidence of all the encountered filarial spp except for *S. equina*, where the infection rate prevailed in males versus females (40.82% vs. 31.11%) (Table-1). In general, There was a significant effect of age on the rate of filarial infection (p< 0.05) which was more prevalent at donkeys 5-15 years of age (61.70%) than those >15 years and < 5 years of age (57.45% and 9.57%, respectively) (Table-2).

**Table-2:** Effect of age on the prevalence of filarial nematodes.

Filarial nematodes	Infection (%)		

	<5 years (n=29)	515 years (n=91)	>15 years (n=68)
*O. cervicalis*	11 (37.93)^aB^	77 (84.62)^aA^	68 (100)^aA^
*O. reticulata*	0 (0)^bA^	3 (3.30)^cA^	5 (7.35)^cA^
*S. equina*	7 (24.14)^abA^	33 (36.26)^bA^	28 (41.18)^bA^
*P. multipapillosa*	0 (0)^bA^	3 (3.30)^cA^	7 (10.29)^cA^
Total (n=188)	18 (9.57)^C^	116 (61.70)^A^	108 (57.45)^B^

Different superscripts letters

(a,b,c) in the same column indicate significant differences at p<0.05. Different superscripts letters

(A,B,C) in the same row indicate significant differences at p<0.05. *O. cervicalis=Onchocerca cervicalis, S. equina=Setaria equina, O. reticulata=Onchocerca reticulata, P. multipapillosa=Parafilaria multipapillosa*

**Figure-2:**
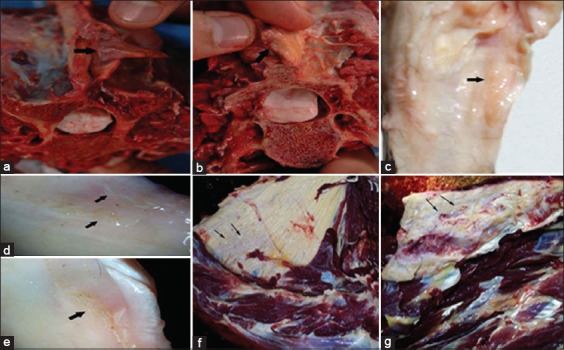
(a and b) *Onchocerca cervicalis* adults in the insertion point of the nuchal ligament (lamellar part); (c-e) *Onchocerca reticulata* adult worms in the flexor and suspensory ligaments of the forelegs; (f,g) Nuchal ligaments suffering from fibrosis and calcification due to *O. cervicalis*.

Filarial worms tended to influence distinctive parts of the body. *O. cervicalis* was confined to the nuchal ligament (86.70%) (Figure-2a and b). *O. reticulata* was found in the suspensory and flexor tendons of the forelegs (4.26%) (Figure-2c-e). *S. equina* occupied the peritoneal and pleural cavities and testes (36.17% and 10%, respectively). *P. multipapillosa* adult worms and microfilariae inhabited the subcutaneous tissues (5.23%). Microfilariae of *O*. *cervicalis* and *O. reticulata* were exhibited in skin scraps and skin snips biopsies got from the midline of the abdomen, particularly umbilical region of 82.45% and 4.26% of donkeys, respectively. However, they were not observed in the blood. Microfilariae of *S. equina* were never detected all through the time of the study (Table-3).

**Table-3:** Frequency of filarial nematodes in different tissues at PM examination.

Filarial nematodes	Infection (%)						

	Adult worms					Microfilariae	
	
	Nuchal ligament (n=188)	S&F tendons (n=188)	P&P cavities (n=188)	Testes (n=150)	Eyes (n=376)	Skin (n=188)	Blood (n=188)
*O. cervicalis*	163 (86.70)	0 (0)	0 (0)	0 (0)	0 (0)	155 (82.45)	0 (0)
*O. reticulata*	0 (0)	8 (4.26)	0 (0)	0 (0)	0 (0)	8 (4.26)	0 (0)
*S. equina*	0 (0)	0 (0)	68 (36.17)	15 (10)	0 (0)	0 (0)	0 (0)
*P. multipapillosa* adult	0 (0)	0 (0)	0 (0)	0 (0)	0 (0)	10 (5.32)	0 (0)

The testis of some donkeys could not be examined because of castration. S&F=Suspensory and flexor tendons of fore legs, P&P=Peritoneal and pleural cavities, *O. cervicalis=Onchocerca cervicalis, S. equina=Setaria equina, O. reticulata=Onchocerca reticulata, P. multipapillosa=Parafilaria multipapillosa*, PM=Post mortem

*O*. *cervicalis* adult was seen entangled in cavities in the nuchal ligament or in the loose connective tissue and adipose tissues surrounding the ligament bringing about serious incendiary tissue sore in the nuchal ligament. The nuchal ligament of tainted donkeys of 5-year-old appeared grossly normal. Elder donkeys over 5-year-old suffered from caseation, mineralization, and granulomatous lesion of the nuchal ligament associated with the death of the parasites. The lesion ranged from focal isolated areas of 2-3 mm in diameter to a complete substitution of the normal ligament tissues (Figure-2f and g). *O. reticulata* was found in the suspensory and flexor tendons of forelegs causing inflammation and edema in place. *S. equina* was extricated from peritoneal and pleural cavities and testes with no observed gross lesion. *P*. *multipapillosa* brought on a hemorrhagic dermatitis with inflammation the subcutaneous and intramuscular tissue accompanied by extensive edema.

## Discussion

The findings that 86.70% out of 188 examined donkeys were infected with filarial nematodes, and *O*. *cervicalis* appeared to be the most common species (82.98%) were consistent with the previous report Bahnass [7] who found filarial worm infestation in 84.5% of donkeys in Sharkia province in Egypt. On the contrary, a lower incidence was recorded by El-Wahab and Raef [8] (65.38%). The incidence of *S. equina* (36.17%) concurred with that reported by Radwan [9]. This result was inconsistent with other previous investigators [10,11]. *P. multipapillosa* was found in 5.32% of the examined donkeys, which agreed with the report of Arafa [12]. Alternately, a higher infection rate was recorded by Maloufi [13]. The lower incidence of *O. reticulata* (4.26%) observed in this study disagreed with the result of Yousif *et al*. [14]. The recorded varieties in the prevalence of filarial nematodes might be credited to the environmental conditions which influence the continuance and transmission flow of the vectors and thus influence the severity and burden of filarial infection or to the degree of attention given by donkey owners to their animal management.

Severe pathological lesion associated with *O. cervicalis* adults in the ligamentum nuchae of elder donkeys was formerly seen by Jubb *et al*. [15]. In spite of the fact that, donkey eyes could be tainted with *S. equina* [16], this was not experienced in this study, where the regular destinations of *S. equina* were peritoneal and pleural cavities and testicles [10]. The intense hemorrhagic lesion that accompanied *P*. *multipapillosa* could be ascribed to the habit of the female of penetrating the skin to lay eggs and larvae [17].

In the present study, no significant effect of the sex on the incidence of all the encountered filarial speciesexcept *S. equina*, which its infection rate prevailed in males versus females (40.82% vs. 31.11%). Furthermore, noticeable variety in filarial predominance was indicated by age, where filarial infection was more prevalent among donkeys of 5-15 years of age. In this respect, the prevalence of filariasis was formerly found to be higher in females than males (43% vs. 31%) and in the age group of 7-16 versus 1-6 years (34.3% vs. 32.3%) [18]. On the other hand, Al Anazi *et al*. [10] found that the age did not have a significant effect on the incidence of *S. equina*.

Microfilariae of the encountered filarial worms were mainly recorded in skin biopsies or skin snips obtained from the midline of the abdomen, especially umbilical region and not perceived in the examined blood samples. In opposition, other researchers could identify blood microfilariae [19]. The difference could be explained by the tendency of *Onchocerca* sp. and *P*. *multipapillosa* females in producing microfilariae which migrate directly toward the skin and utilize umbilical region as predilection site [20]. Similar results were previously noticed by several researchers [21]. From our perspective, the absence of microfilariae in *S. equina* may be ascribed to the low level of microfilariae which make them difficult to be detected upon examination or to the high immunity of the host that restricts the circulation of microfilariae.

## Conclusion

These preliminary results add to our understanding of filarial infection in donkeys in Egypt, their impact on animal performance and production. Accordingly, emphasis must be taken for prevention, control of filarial infection, and improvement of the management system of donkeys.

## Authors’ Contributions

Authors NEA and LME designed and conducted the study. AMR and MYR contributed in sample collection and application of the laboratory analysis. RSE contributed in collecting of samples and review of the literatures, analyzing data, drafting and revising the manuscript. All authors read and approved the final manuscript.
